# Navicular Stress Fractures: A Narrative Review of Pathoanatomy, Diagnostic Pitfalls, and Management

**DOI:** 10.7759/cureus.97078

**Published:** 2025-11-17

**Authors:** Zubair Younis, Balu Ravi, Karim Rezk, Jay Shah, Muhammad A Hamid

**Affiliations:** 1 Orthopaedics, The Royal Wolverhampton NHS Trust, Wolverhampton, GBR; 2 Trauma and Orthopaedics, The Royal Wolverhampton NHS Trust, Wolverhampton, GBR; 3 Trauma and Orthopaedics, Airedale NHS Foundation Trust, West Yorkshire, GBR; 4 General Surgery, The Royal Wolverhampton NHS Trust, Wolverhampton, GBR; 5 Orthopaedic Surgery, University Hospitals Birmingham, Birmingham, GBR

**Keywords:** bone healing, computed tomography, internal fixation, midfoot pain, navicular stress fracture, nonunion, overuse injury

## Abstract

Navicular stress fractures are high-risk overuse injuries of the midfoot that most commonly affect athletes, military recruits, and individuals involved in repetitive weight-bearing activities, with an incidence ranging from 0.7% to 35% in different studies. Although uncommon in the general population, they pose significant diagnostic and therapeutic challenges due to their tendency for delayed union, nonunion, and chronic pain. The unique anatomy of the tarsal navicular, functioning as the keystone of the medial longitudinal arch, exposes it to considerable mechanical stress, while its central watershed zone predisposes it to relative avascularity and impaired healing. Patients typically present with vague, activity-related midfoot pain and focal tenderness over the dorsal proximal navicular, often leading to prolonged diagnostic delays. Plain radiographs frequently appear normal in the early stages, necessitating the use of advanced imaging such as computed tomography and magnetic resonance imaging for accurate detection and classification. Management depends on the fracture type, degree of displacement, and patient activity level. Nondisplaced fractures generally respond well to strict non-weight-bearing immobilization, while displaced or nonunited fractures often require surgical fixation to achieve predictable union and earlier return to activity. Operative management has been associated with higher success rates, lower refracture risk, and faster functional recovery in high-demand individuals. Adjunctive modalities such as bone stimulators, shockwave therapy, and biologic augmentation with bone graft or bone marrow aspirate are increasingly explored to enhance healing, though evidence remains limited. Successful rehabilitation emphasizes a gradual return to weight-bearing, correction of biomechanical risk factors, and optimization of bone health. Early recognition, appropriate imaging, and individualized management strategies remain essential to achieving favorable outcomes. Further research should focus on standardized diagnostic pathways, prospective comparative studies, and the role of biologic and imaging adjuncts in improving union rates and return-to-sport timelines.

## Introduction and background

Navicular stress fractures (NSFs) are a significant and often underdiagnosed overuse injury of the midfoot. While first described in the athletic population, they are increasingly recognized in a diverse range of individuals engaged in repetitive weight-bearing activities, including military recruits, dancers, and occupationally active persons [1,2]. Although relatively rare in the general sedentary population, NSFs represent a substantial diagnostic and therapeutic challenge due to their propensity for delayed union, nonunion, and chronic pain if not managed appropriately [3]. They account for a notable proportion of all stress fractures in the foot and ankle, with reported incidences ranging from 0.7% to as high as 35% in certain high-risk groups [2,3].

**Figure 1:**
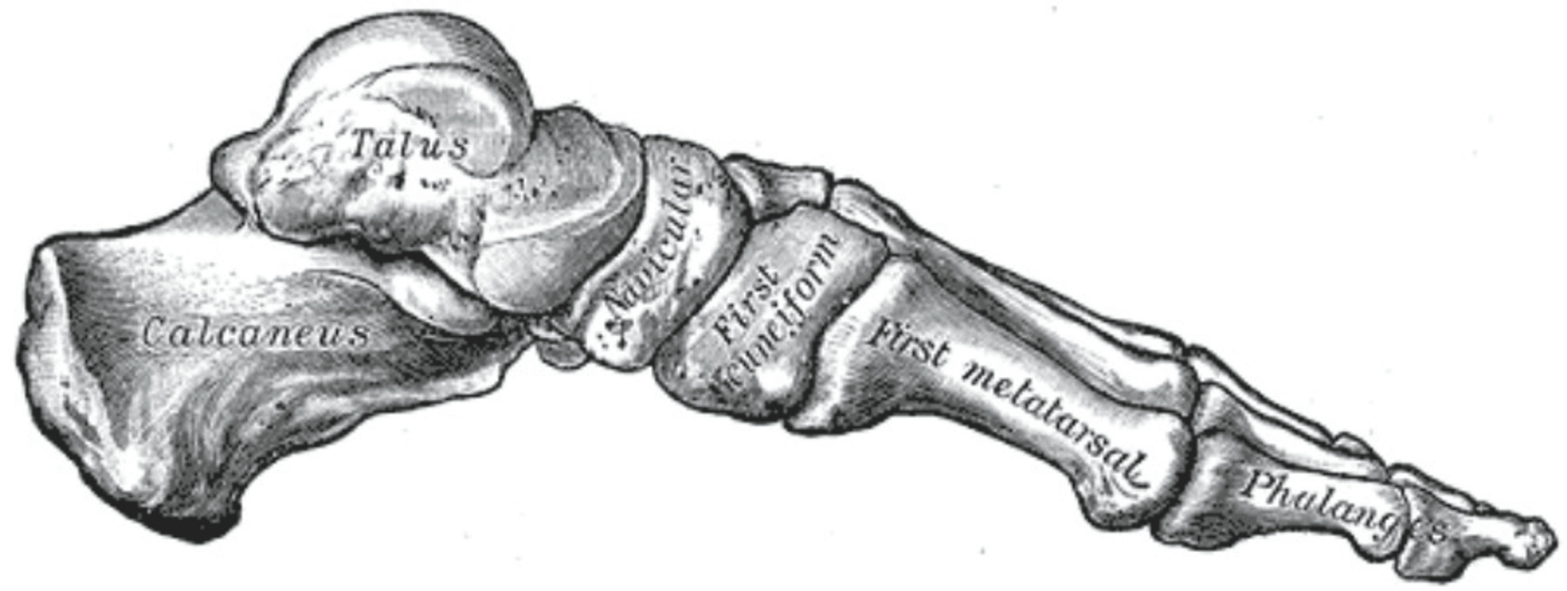
Schematic illustration of the navicular as the cornerstone of the medial longitudinal arch Image Credits: Wikipedia (https://upload.wikimedia.org/wikipedia/commons/8/81/Gray290.png) This work is in the public domain in the United States because it was published (or registered with the U.S. Copyright Office) before January 1, 1930.

The pathoanatomy of the tarsal navicular bone itself is central to its vulnerability. As the keystone of the medial longitudinal arch, the navicular is critical for force transmission between the hindfoot and forefoot (Figure 1) [4]. Its unique saddle shape and its articulation with the talus and cuneiforms subject it to significant biomechanical stress. Compounding this, the vascular supply to the central third of the bone is a watershed area, receiving branches from the dorsalis pedis and posterior tibial arteries that enter medially and laterally, leaving the central portion relatively avascular [4-6]. This confluence of high mechanical stress and compromised blood flow creates an environment ripe for the development of microfractures and impaired healing [5,7].

Clinically, NSFs are notorious for their insidious and vague presentation. Patients often report ill-defined, aching midfoot pain that is exacerbated by activity and relieved by rest [1,8]. The pathognomonic "N-spot" tenderness on the proximal dorsal aspect of the navicular is a key physical finding but is frequently missed, contributing to a well-documented delay in diagnosis that can average from four months to over a year [1,8]. Detailed patient history and a thorough physical examination can play an important role in diagnosing NSF. Initial plain radiographs are normal in the majority of cases, necessitating a high index of suspicion and the use of advanced imaging such as computed tomography (CT) or magnetic resonance imaging (MRI) for confirmation [9,10].

The management paradigm for NSFs has evolved considerably. Traditional non-operative management with prolonged non-weight-bearing cast immobilization remains a cornerstone, particularly for nondisplaced fractures [1,9]. However, a growing body of evidence, including a recent meta-analysis, highlights that operative intervention may offer superior outcomes in terms of success rates, lower refracture risk, and potentially faster recovery for specific fracture types and in patients for whom prolonged immobilization is not feasible [4,10]. This narrative review aims to synthesize the current evidence on NSFs in athletes and the general population, providing a comprehensive overview of their etiology, diagnostic workflow, and the spectrum of contemporary management strategies tailored to the individual patient's needs and demands. 

## Review

Epidemiology

NSFs are considered a high-risk stress fracture due to their potential for delayed union, nonunion, and prolonged disability [1]. Historically perceived as rare, their reported incidence has increased with greater clinical awareness and the widespread availability of advanced imaging modalities like CT and MRI [2]. While they represent a small fraction (0.7-2.4%) of all stress fractures in some general studies, this figure rises dramatically to 14-35% in studies focusing on the foot and ankle and in athletic cohorts [2,3].

The demographic most commonly affected is young, active adults. A large review of 200 cases found the majority occurred in males in their mid-twenties, with over 98% being athletes due to their engagement in high-impact, repetitive loading sports that concentrate stress on the relatively avascular central third of the navicular [1]. However, this should not obscure their occurrence in other populations. Military recruits, who engage in intense, repetitive weight-bearing exercises, are another well-documented group [5]. Furthermore, NSFs are reported in dancers, gymnasts, and individuals whose occupations involve prolonged standing or walking on hard surfaces [4,6]. Anatomical variations such as pes cavus, a short first metatarsal, metatarsus adductus, and limited ankle dorsiflexion have been implicated as intrinsic risk factors, though no single factor has demonstrated definitive statistical significance across all studies [4,7]. Extrinsic factors, including training errors (a sudden increase in activity intensity or duration), improper footwear, and poor nutritional status (particularly vitamin D deficiency), are also significant contributors across all patient groups [1,11].

A critical and consistent feature in the epidemiology of NSFs is the marked delay in diagnosis, which averages from four to over nine months from symptom onset [3,10]. This delay is multifactorial, stemming from the injury's insidious presentation, the lack of sensitivity of initial radiographs, and a low index of suspicion among clinicians, often leading to initial misdiagnoses such as midfoot sprain or tendonitis [4,12]. This protracted period from symptom onset to definitive diagnosis can adversely impact healing potential and overall patient outcomes.

Anatomy and pathoanatomy

A thorough understanding of the osseous, articular, and vascular anatomy of the tarsal navicular is essential to comprehend its susceptibility to stress fractures.

Osseous and Articular Anatomy

The tarsal navicular is a critical cornerstone of the medial longitudinal arch of the foot. It is a flattened, concave, boat-shaped bone wedged between the head of the talus proximally and the three cuneiform bones distally [4,12]. Its proximal concave articulation with the talar head forms the talonavicular joint, a key component of the midtarsal (Chopart) joint that allows for significant inversion and eversion [2]. Distally, it has three separate articular facets for the medial, intermediate, and lateral cuneiforms, creating a rigid midfoot construct known as the Lisfranc joint complex [13]. Medially, the navicular tuberosity serves as the primary insertion site for the posterior tibial tendon, a major dynamic stabilizer of the arch [4].

Vascular Anatomy

The vascular supply to the navicular is precarious and is central to the pathoanatomy of stress fractures. The bone is largely covered by articular cartilage, limiting the entry points for blood vessels. Its blood supply is derived from branches of the dorsalis pedis artery dorsally and the posterior tibial artery plantarly and at the tuberosity [6,13]. These vessels anastomose within the bone, radiating from the non-articular surfaces toward the center. Classic studies by Torg et al. and subsequent cadaveric investigations have confirmed that this vascular pattern results in a watershed area of relative avascularity in the central third of the navicular [5].

Biomechanics and Pathophysiology

The navicular is subjected to significant shear and compressive forces during gait. During foot strike and push-off, it becomes maximally impinged between the talus and the cuneiforms [4,12]. Biomechanical analyses indicate that the majority of this force is concentrated at the central one-third of the bone, which coincides precisely with the watershed avascular zone [14]. This creates a critical area of high mechanical stress and low healing potential. Repetitive cyclic loading, especially in the presence of antagonistic muscular pull from the posterior tibial tendon, eventually leads to cumulative microdamage that outpaces the body's reparative capacity [4,7]. This continuum begins as a "stress reaction" (bone edema visible on MRI) and progresses to a cortical break, a true stress fracture typically initiating in the proximal dorsal cortex and propagating plantarwards into the navicular body [2,10]. This confluence of unique biomechanical stress and tenuous vascularity is what classifies the navicular as a high-risk site for stress fractures. There are potential consequences of untreated stress fractures, such as prolonged recovery time or increased risk of complications like delayed union, nonunion, avascular necrosis, secondary arthritis, midfoot instability, and stiffness.

Risk factors

The development of an NSF is typically multifactorial, resulting from an imbalance between bone stress and bone remodeling capacity. The risk factors can be broadly categorized into intrinsic (patient-specific) and extrinsic (environmental/activity-related) factors [1].

Intrinsic Risk Factors

Anatomic variations that alter foot biomechanics are significant contributors.

Foot structure: Pes cavus (a high-arched foot) is frequently implicated due to its inherent rigidity, and poor shock absorption can result in increased medial midfoot stress during forefoot strike and push-off [2,12]. Other variants, such as metatarsus adductus, a short first metatarsal, and a long second metatarsal, can increase shear stress across the central navicular by altering force transmission through the midfoot and predisposing to microfracture [4,5].

Joint mobility: Limited ankle dorsiflexion and restricted subtalar motion can compromise the foot's ability to dissipate forces, leading to compensatory overload at the navicular [1,7].

Biological factors: Nutritional deficiencies, particularly of vitamin D and calcium, are critical risk factors that impair bone health and healing capacity [1,11]. In females, components of the female athlete triad (low energy availability, menstrual dysfunction, and low bone mineral density) significantly increase the risk for all bony stress injuries, including NSFs [15]. A genetic predisposition has also been suggested by case reports of NSFs in monozygotic twins [16].

Extrinsic Risk Factors

These factors relate to the external demands placed on the bone.

Training errors: A sudden increase in the intensity, duration, or frequency of activity is the most common extrinsic trigger. This is often seen in new military recruits, athletes beginning a new season, or individuals starting a new exercise regimen [2,10].

Equipment and environment: Inappropriate or worn-out footwear that provides inadequate support or shock absorption can contribute to NSF development. Training on hard or uneven surfaces also increases impact forces [1,7].

Occupational demands: Occupations that require prolonged standing, walking, or heavy lifting can provide the repetitive cyclical loading necessary to precipitate a stress fracture, even in non-athletic individuals [4].

Clinical presentation

Patients with an NSF typically present with an insidious onset of poorly localized pain in the dorsal midfoot, which may radiate along the medial longitudinal arch [4,17]. The history is often more revealing than the physical examination. The pain is characteristically activity-related, initially appearing at the end of a period of exertion and resolving with rest. As the stress injury progresses, the pain occurs earlier during activity and may persist for hours or even days after cessation [2,10]. In later stages, pain can be present during routine weight-bearing activities like walking.

Physical Examination

Findings on physical exam are often subtle, which contributes to diagnostic delay. The most reliable sign is pinpoint tenderness over the "N-spot," a nickel-sized area on the proximal dorsal aspect of the navicular bone. The N-spot is located at the central one-third of the dorsal navicular, approximately 2 cm distal to the tip of the medial malleolus and just lateral to the tibialis anterior tendon. It lies between the tibialis anterior and extensor hallucis longus tendons, over the midpoint of the navicular body. Studies indicate this is present in over 80% of confirmed cases with a reported sensitivity of 81-100% and a specificity of about 84% when correlated with CT or MRI findings [5,10]. Pain can often be elicited by having the patient perform a single-leg heel raise or hop on the affected foot. Pain with standing on the toes in the equinus position is another common finding [7,18]. Notably, there is typically an absence of significant swelling, ecchymosis, or deformity. The neurovascular examination is almost always normal, and patients usually have a full range of motion at the ankle and subtalar joints, though some may exhibit limited dorsiflexion [4,17]. Due to this vague and often unimpressive clinical picture, NSFs are frequently misdiagnosed initially as midfoot sprains, posterior tibial tendonitis, or plantar fasciitis. A high index of suspicion is therefore paramount in any active individual presenting with unexplained, persistent midfoot pain.

Diagnostic workup

The diagnostic workup for an NSF requires a high index of suspicion, as reliance on clinical examination and initial radiographs alone often leads to misdiagnosis. A systematic approach to imaging is crucial for confirmation and treatment planning.

Plain Radiography

Weight-bearing anteroposterior, lateral, and oblique views of the foot are typically the first-line imaging studies. However, their utility is limited by poor sensitivity, which is reported to be as low as 33% [4]. However, its limited sensitivity, radiographs remain useful for excluding other causes like an accessory navicular. Radiographs are often normal in the early stages, as visible fracture lines or periosteal reaction require significant bone resorption, a process that can take two to three weeks or more [2]. While a positive radiograph is helpful for diagnosis, a negative study cannot rule out an NSF. Their primary value lies in excluding other bony pathologies such as fractures, osteoarthritis, or accessory navicular syndromes [1,4]. Due to the limitations of radiography, advanced imaging is almost always necessary for a definitive diagnosis.

Bone Scintigraphy (Bone Scan)

A triple-phase bone scan is highly sensitive (approaching 100%) and can be positive within 48-72 hours of symptom onset [4,19]. It demonstrates increased radiotracer uptake in all three phases within the entire navicular. However, it lacks specificity, as it cannot differentiate a stress fracture from a stress reaction, infection, or other bone pathologies. It also provides poor anatomic detail regarding fracture morphology, displacement, or comminution [19].

Computed Tomography

CT scanning is considered the gold standard for characterizing a confirmed NSF [20]. It provides excellent bony detail, allowing for clear visualization of the fracture line, assessment of completeness, displacement, comminution, and the presence of sclerosis or cystic changes at the fracture margins, all critical factors for classification and guiding management [9,21]. Thin-slice (1-1.5 mm) coronal and axial reconstructions are optimal for evaluation.

Magnetic Resonance Imaging

MRI is exquisitely sensitive for detecting early bone marrow edema, which represents a "stress reaction" before a cortical fracture line develops [1,22]. It is therefore the preferred modality when clinical suspicion is high but the CT scan is negative or equivocal. MRI offers excellent soft tissue and bony visualization without radiation exposure and can delineate associated injuries. On MRI, an NSF appears as a linear hypointense fracture line on T1-weighted images surrounded by bone marrow edema (hyperintense on T2-weighted or short tau inversion recovery (STIR) sequences) [22].

Classification

The most clinically utilized classification system for NSFs is the CT-based system proposed by Saxena et al., which correlates with prognosis and can guide treatment decisions (Table 1) [9].

**Table 1:** Computed tomography-based classification of navicular stress fractures

Type	Fracture description		Clinical significance/prognosis	
Type I	Fracture involves only the dorsal cortex of the navicular.		Incomplete fracture; usually heals well with 6-8 weeks of non-weight-bearing immobilization. Excellent prognosis with conservative management.	
Type II	Fracture extends from the dorsal cortex into the body of the navicular, but not to the opposite cortex.		Partial fracture; longer healing time. May still respond to non-operative care, but surgical fixation is often considered for athletes.	
Type III	Fracture propagates into a second cortex (plantar, medial, or lateral).		Complete bicortical fracture; higher risk of delayed or nonunion. Surgical fixation is typically required, especially in high-demand patients.	

The system also includes modifiers for complicating features: A for avascular necrosis, C for cystic changes, and S for sclerosis at the fracture site. This classification has prognostic value, with type III fractures and those with sclerotic modifiers generally requiring a longer healing time and being more prone to nonunion, often necessitating surgical intervention [9].

It is also important to contextualize NSFs within the broader framework of stress injuries. The Boden classification categorizes stress fractures as "high-risk" or "low-risk" based on their inherent healing potential [23]. Due to its tenuous vascularity and high mechanical stress, the navicular is unequivocally classified as a high-risk stress fracture, indicating a propensity for delayed union, nonunion, and a requirement for more aggressive treatment [1,23].

Differential diagnosis

The vague presentation of midfoot pain necessitates considering several other conditions.

Midfoot Sprain (Lisfranc Injury)

Often presents with midfoot pain and swelling. Pain is typically elicited with direct palpation of the Lisfranc joint and with stress testing (pronation-abduction of the forefoot), unlike the localized dorsal "N-spot" tenderness of an NSF [4].

Posterior Tibial Tendon Dysfunction

It can cause medial midfoot pain. However, pain is typically located posterior to the medial malleolus and along the tendon's course, with possible associated loss of strength (difficulty with single-heel rise) and acquired flatfoot deformity [4].

Plantar Fasciitis

It is characterized by sharp, stabbing pain in the plantar heel, typically worst with the first steps in the morning. Pain is localized to the medial calcaneal tubercle, not the dorsal navicular [2].

Accessory Navicular Syndrome

Pain arises from a secondary ossification center at the navicular tuberosity, exacerbated by pressure from footwear and resisted foot inversion. Tenderness is medial, not dorsal [4].

Kohler's Disease

Idiopathic osteonecrosis of the navicular typically affects children aged four to seven years. Radiographs show sclerosis and flattening of the navicular bone [5].

Degenerative Arthritis of the Talonavicular or Midfoot Joints

Pain is typically activity-related and associated with joint stiffness. Radiographs may show joint space narrowing and osteophyte formation [2].

Morton's Neuroma

It causes sharp, burning pain in the forefoot, often with associated numbness or tingling, and is typically located in the third web space [4].

Management

The management of NSFs is tailored to the fracture characteristics, particularly their completeness and displacement as defined by the Saxena classification, and the functional demands of the patient. The primary goals are to achieve fracture union, prevent nonunion or refracture, and facilitate a safe return to activity.

Non-operative Management

Non-operative management is the initial treatment of choice for incomplete, nondisplaced fractures (Saxena type I and some type II) and for lower-demand patients [1,10]. However, the decision to pursue a conservative path, especially in high-demand athletes, requires a careful weighing of its potential benefits against its significant risks. A high index of suspicion and early advanced imaging (MRI/CT) are emphasized because diagnosis is commonly delayed, and delays worsen outcomes [24]. The core risk, particularly for the competitive athlete, is the potential for failure, resulting in a delayed union or nonunion; this translates to a significant loss of time and conditioning due to the prolonged strict non-weight-bearing period, and a failed conservative treatment means the athlete endures this lengthy immobilization only to potentially require surgery later, effectively doubling their total time away from sport.

Core protocol: The cornerstone of conservative treatment is strict non-weight-bearing (NWB) immobilization in a cast or controlled ankle movement (CAM) walker boot for a period of six to eight weeks [10]. This is critical to eliminate the shear forces across the fracture site and allow healing. Studies have shown significantly poorer outcomes with weight-bearing casts or simple activity modification, with one landmark study reporting a cure rate of only 26% with activity limitation alone [5]. Studies stress that a hard cast is typical but may cause “cast disease” (calf atrophy and stiffness) in high-level athletes; therefore, a removable boot is often preferred for athletes to preserve ankle range of motion while maintaining NWB compliance [24].

Rehabilitation: After the initial immobilization period, patients are reassessed. The absence of tenderness at the "N-spot" marks the beginning of a graduated functional rehabilitation program [2,10]. This typically involves several weeks of progressive weight-bearing in a boot, followed by physical therapy focusing on restoring range of motion, strength, and proprioception. Low-impact cross-training (e.g., swimming and cycling) is permitted before a gradual, pain-guided return to running and sport-specific activities, which typically takes an additional two to three months [10]. Timing to return to sport varies by fracture type (Saxena): type I ≈3.0 months, type II ≈3.6 months, and type III ≈6.8 months in published series, useful benchmarks when counselling patients [25].

Adjunctive therapies: The use of adjunctive modalities such as bone stimulators, nutritional supplementation, and pharmacological agents has been explored to enhance healing in NSFs. Bone stimulators, including low-intensity pulsed ultrasound and capacitive coupling devices, are commonly employed, though high-quality evidence supporting their efficacy specifically for NSFs remains limited. Their use is considered reasonable to potentially accelerate healing in high-demand individuals [1,26]. Extracorporeal shockwave therapy (ESWT) has also emerged as a promising, noninvasive adjuvant, and authors recommend considering ESWT for both surgically and nonsurgically managed patients where feasible [24]. Nutritional optimization, particularly through correcting vitamin D deficiency and ensuring adequate calcium intake, is strongly advised, as hypovitaminosis D is prevalent among patients with stress fractures and can significantly impair bone healing [1,11]. In cases of delayed healing or nonunion, anabolic agents such as teriparatide (recombinant parathyroid hormone) have demonstrated potential in stimulating bone formation in small studies, though their use remains off-label for this indication. While direct evidence for teriparatide in navicular fractures is limited, animal and small human trials suggest it may benefit difficult-to-heal stress fractures and delayed unions [1].

Operative Management

Surgical intervention is indicated for displaced fractures, fractures that fail an adequate trial (≥3 months) of conservative management, and in high-demand athletes seeking a more predictable and potentially faster return to activity [8,9]. A 2021 meta-analysis demonstrated that operative management provides a significantly higher success rate (97.9% vs. 78.1%) and a lower refracture rate compared to conservative care [3]. Recent reviews advocate earlier surgical consideration in athletes with Saxena type II-III fractures to shorten time lost from sport and improve union rates [25].

Indications: Surgical management is indicated for Saxena type III (bicortical) fractures, displaced fractures, and those with cystic changes or significant sclerosis at the fracture margins. It is also recommended in cases of nonunion or delayed union, as well as failure of non-operative management [8,9,21]. In athletic populations, some authors advocate for surgical fixation of both primary and recurrent NSFs, with the exception of isolated MRI-only stress reactions, to facilitate earlier return to sport and reduce the risk of recurrence [25].

Surgical techniques: The principle of surgery is to achieve stable internal compression across the fracture site and, if needed, to promote biological healing. For nondisplaced or minimally displaced fractures, percutaneous screw fixation with one or two partially or fully threaded cannulated screws placed from lateral to medial is the preferred technique. This minimizes soft tissue dissection and preserves the tenuous blood supply [8]. Intraoperative three-dimensional imaging (O-arm/intraop CT) can improve the accuracy of guidewire and screw placement and reduce malposition, and the authors describe routine use where available [24]. For displaced fractures or nonunions, open reduction and internal fixation (ORIF) using an open dorsal approach is performed. The fracture site is debrided of fibrous tissue, and the sclerotic edges are drilled to stimulate healing. Stable compression is achieved with screws [21]. When opening the fracture site, the authors recommend debridement, drilling of sclerotic margins, and augmentation with biologics as indicated [24]. Bone grafting using autologous bone graft, typically harvested from the iliac crest, is often used to fill defects and augment biology in cases of nonunion, cystic changes, or when a gap remains after debridement [1,21]. Patel et al. describe a technique of iliac crest bone marrow aspirate plus autograft packed into the navicular and recommend this biologic augmentation in athletes and nonunions [24]. Vascularized bone graft is reserved for recalcitrant nonunions or osteonecrosis. Adjuncts at fixation include bone marrow aspirate concentrate (BMAC) delivered via the cannulated screw tract and autograft sleeves, which are described as routine adjuncts in the authors’ preferred technique [24].

Postoperative rehabilitation: The postoperative protocol is similar to the later stages of non-operative care. Patients are typically kept non-weight-bearing for four to six weeks postoperatively, followed by protected weight-bearing in a boot and a gradual, phased return to activity over four to six months [8,10]. Healing is often confirmed with a CT scan prior to clearing a patient for full, high-impact activities. Patel et al. review recommends scheduled CT follow-up (authors commonly obtain CT at ~6 and ~10 weeks) to document progressive consolidation and confirm healing before return-to-play clearance [24].

Return to Activity and Outcomes

The timeline for return to full activity varies significantly between individuals and treatment paths. With successful non-operative management, return to sport typically averages five to six months [5,10]. Following surgical intervention, return to play may be possible around four to five months, with some studies reporting a mean of 4.2 months [3]. Studies show earlier return in surgically treated athletes for type II/III injuries compared with conservative care in some cohorts, supporting early fixation for elite athletes who require predictable, faster return [24,25].

Regardless of the treatment modality, clearance should be based on clinical examination (absence of tenderness) and radiologic confirmation of union on CT scan [21]. Premature return to activity is a major risk factor for refracture. A comprehensive approach that includes addressing underlying biomechanical risk factors with orthotics and correcting training errors is essential for preventing recurrence [1,10]. Because navicular fractures are often related to unique biomechanics and variable vascularity of the central navicular, thorough assessment (gait, footwear, training load, ankle dorsiflexion, and forefoot morphology) and targeted correction are emphasized [24].

Future directions

Despite significant advances in imaging and fixation techniques, the current evidence base for NSFs remains limited, with most studies being small case series or retrospective reviews [3]. Both historical and contemporary literature highlight persistent heterogeneity in diagnostic criteria, imaging protocols, and rehabilitation strategies, which hampers the formulation of standardized treatment algorithms [24].

Future research should prioritize prospective, multicenter studies comparing modern operative fixation with optimized non-operative protocols, stratified by fracture type and athletic demand [21]. Standardizing definitions of healing (clinical and CT-based) and functional outcomes would improve comparability across studies [25].

Advances in imaging offer an opportunity to enhance diagnostic precision. Weight-bearing and high-resolution CT can better characterize early cortical disruption and guide individualized load management, while intraoperative 3D imaging can improve fixation accuracy [24]. Rigorous evaluation of these technologies in prospective trials is warranted.

The biologic augmentation of fracture healing represents another promising frontier [1]. Future research should prioritize controlled trials to evaluate the efficacy of emerging adjuvants like BMAC, platelet-rich plasma (PRP), and ESWT specifically for NSFs [27]. Although autograft, BMAC, and shockwave therapy are increasingly used in elite athletes, their true efficacy remains unproven and requires controlled, comparative studies [24].

Finally, long-term outcome studies are essential to clarify the risks of refracture and post-traumatic arthrosis. Establishing a centralized registry of NSFs could facilitate such surveillance and guide evidence-based recommendations.

## Conclusions

NSFs represent a complex and often under-recognized cause of midfoot pain, particularly in athletes and active individuals. Their subtle presentation, coupled with the limitations of plain radiography, frequently leads to diagnostic delays that can adversely affect outcomes. A high index of suspicion, early use of advanced imaging, including MRI, and timely intervention are essential for optimal recovery. Conservative management remains effective for nondisplaced fractures when strict non-weight-bearing is maintained, while surgical fixation offers superior results in displaced, chronic, or high-demand cases. Successful treatment also relies on addressing contributing factors such as training errors, biomechanical abnormalities, and nutritional deficiencies. Multidisciplinary involvement, combining orthopedic, physiotherapy, and sports medicine expertise, ensures a structured rehabilitation and safe return to activity. As our understanding of NSFs continues to evolve, there is a growing need for standardized diagnostic algorithms and robust clinical trials to refine treatment strategies. Ultimately, individualized care guided by early diagnosis, anatomical understanding, and evidence-informed management remains the cornerstone of achieving durable union and preventing recurrence.

## References

[1] Shakked RJ, Walters EE, O’Malley MJ (2017). Tarsal navicular stress fractures. Curr Rev Musculoskelet Med.

[2] Lee S, Anderson RB (2004). Stress fractures of the tarsal navicular. Foot Ankle Clin.

[3] Attia AK, Mahmoud K, Bariteau J, Labib SA, DiGiovanni CW, D’Hooghe P (2021). Return to sport following navicular stress fracture: a systematic review and meta-analysis of three hundred and fifteen fractures. Int Orthop.

[4] Coris EE, Lombardo JA (2003). Tarsal navicular stress fractures. Am Fam Physician.

[5] Torg JS, Pavlov H, Cooley LH, Bryant MH, Arnoczky SP, Bergfeld J, Hunter LY (1982). Stress fractures of the tarsal navicular. A retrospective review of twenty-one cases. J Bone Joint Surg Am.

[6] McKeon KE, McCormick JJ, Johnson JE, Klein SE (2012). Intraosseous and extraosseous arterial anatomy of the adult navicular. Foot Ankle Int.

[7] Fitch KD, Blackwell JB, Gilmour WN (1989). Operation for non-union of stress fracture of the tarsal navicular. J Bone Joint Surg Br.

[8] Saxena A, Fullem B (2006). Navicular stress fractures: a prospective study on athletes. Foot Ankle Int.

[9] Saxena A, Fullem B, Hannaford D (2000). Results of treatment of 22 navicular stress fractures and a new proposed radiographic classification system. J Foot Ankle Surg Off Publ Am Coll Foot Ankle Surg.

[10] Khan KM, Brukner PD, Kearney C, Fuller PJ, Bradshaw CJ, Kiss ZS (1994). Tarsal navicular stress fracture in athletes. Sports Med.

[11] Gorter EA, Hamdy NAT, Appelman-Dijkstra NM, Schipper IB (2014). The role of vitamin D in human fracture healing: a systematic review of the literature. Bone.

[12] Hossain M, Clutton J, Ridgewell M, Lyons K, Perera A (2015). Stress fractures of the foot. Clin Sports Med.

[13] Waugh W (1958). The ossification and vascularisation of the tarsal navicular and their relation to Köhler’s disease. J Bone Joint Surg Br.

[14] Kitaoka HB, Luo ZP, An KN (1996). Contact features of the talonavicular joint of the foot. Clin Orthop.

[15] Barrack MT, Gibbs JC, De Souza MJ (2014). Higher incidence of bone stress injuries with increasing female athlete triad-related risk factors: a prospective multisite study of exercising girls and women. Am J Sports Med.

[16] Van Meensel AS, Peers K (2010). Navicular stress fracture in high-performing twin brothers: a case report. Acta Orthop Belg.

[17] Gross CE, Nunley JA 2nd (2015). Navicular stress fractures. Foot Ankle Int.

[18] Bennell K, Matheson G, Meeuwisse W, Brukner P (1999). Risk factors for stress fractures. Sports Med Auckl NZ.

[19] Pavlov H, Torg JS, Freiberger RH (1983). Tarsal navicular stress fractures: radiographic evaluation. Radiology.

[20] Kiss ZS, Khan KM, Fuller PJ (1993). Stress fractures of the tarsal navicular bone: CT findings in 55 cases. AJR Am J Roentgenol.

[21] McCormick JJ, Bray CC, Davis WH, Cohen BE, Jones CP 3rd, Anderson RB (2011). Clinical and computed tomography evaluation of surgical outcomes in tarsal navicular stress fractures. Am J Sports Med.

[22] Ariyoshi M, Nagata K, Kubo M (1998). MRI monitoring of tarsal navicular stress fracture healing-a case report. Kurume Med J.

[23] Boden BP, Osbahr DC (2000). High-risk stress fractures: evaluation and treatment. J Am Acad Orthop Surg.

[24] Patel KA, Christopher ZK, Drakos MC, O’Malley MJ (2021). Navicular stress fractures. J Am Acad Orthop Surg.

[25] Jones MH, Amendola AS (2006). Navicular stress fractures. Clin Sports Med.

[26] Beck BR, Matheson GO, Bergman G (2008). Do capacitively coupled electric fields accelerate tibial stress fracture healing? A randomized controlled trial. Am J Sports Med.

[27] Miller TL, Kaeding CC, Rodeo SA (2020). Emerging options for biologic enhancement of stress fracture healing in athletes. J Am Acad Orthop Surg.

